# Domestic laundering of healthcare textiles: Disinfection efficacy and risks of antibiotic resistance transmission

**DOI:** 10.1371/journal.pone.0321467

**Published:** 2025-04-30

**Authors:** Caroline Cayrou, Katie Silver, Lucy Owen, Jake Dunlop, Katie Laird

**Affiliations:** Infectious Disease Research Group, Leicester School of Pharmacy, De Montfort University, Leicester, United Kingdom; Universidad Autonoma de Chihuahua, MEXICO

## Abstract

Hospital-acquired infections (HAIs) and antimicrobial resistance (AMR) are a major public health concern, with the evidence base for the potential role of textiles as fomites in microbial transmission growing. In the UK, domestic laundering machines (DLMs) are commonly used to clean healthcare worker uniforms, raising concerns about their effectiveness in microbial decontamination and role in AMR development. This study aimed to evaluate DLMs’ ability to decontaminate microorganisms and their potential impact on AMR. The performance of six DLMs was assessed using *Enterococcus faecium* bioindicators under various wash cycles and detergent conditions. Shotgun metagenomics was used to analyse the microbiome and resistome of DLMs. The minimum inhibitory concentrations of domestic detergents were determined for *Staphylococcus aureus*, *Klebsiella pneumoniae*, and *Pseudomonas aeruginosa*, and detergent tolerance and antibiotic cross-resistance were assessed. Results showed only 50% (3/6) of DLMs achieved sufficient decontamination (≥5 log10 CFU reduction) at 60°C during full-length cycles, with rapid cycles performing inconsistently. Microbiome analysis revealed the presence of potentially pathogenic bacteria (e.g., *Mycobacterium* sp. *Pseudomonas* sp. and *Acinetobacter* sp.) and antibiotic resistance genes, including efflux pumps and target modification genes. Detergent tolerance assays showed increased bacterial tolerance to detergents, with cross-resistance to antibiotics observed in *S. aureus* and *K. pneumoniae*, including carbapenem and β-lactam groups. Whole genome sequencing identified mutations in genes encoding efflux pumps in *S. aureus* (*MrgA*) and *K. pneumoniae* (*AcrB*) after detergent exposure, which could impact efflux pump function. Findings suggest domestic laundering of healthcare uniforms may be insufficient for decontamination, posing risks for HAI transmission and AMR. Revising laundering guidelines to ensure effective DLM performance, detergent efficacy, and considering alternatives like onsite/industrial laundering are crucial to enhancing patient safety and controlling AMR in healthcare settings.

## Introduction

Hospital-acquired infections (HAIs) are a common complication of hospitalisation, posing significant risks to patient safety by increasing both morbidity and mortality. Reducing the risk of HAIs is especially important considering the fact that antibiotic-resistant microorganisms are frequently associated with HAIs. Six major antibiotic-resistant bacterial species responsible of nosocomial infections have been considered as a priority or critical by the World Health Organisation [1]. These ESKAPE pathogens which refer to: *Enterococcus faecium*, *Staphylococcus aureus*, *Klebsiella pneumoniae*, *Acinetobacter baumanii*, *Pseudomonas aeruginosa* and *Enterobacter* species are often detected in healthcare settings and associated with outbreaks of disease [2].

In order to limit the risk of HAIs, infection control measures are in place within hospital environments, these particularly focus on hard surface and hand disinfection. However, there is less focus on the disinfection of healthcare textiles, which can also be a source infection. Studies have demonstrated that *P. aeruginosa*, *Escherichia coli*, *E. faecium* and *S. aureus* were able to survive at least 20 days on cotton and *E. faecium* and *S. aureus* were still viable after 7 days on polyester [3,4]. Several cases of HAIs associated with healthcare textiles have been reported, for instance, in 2002, an outbreak of Carbapenem resistant *Acinetobacter baumanii* strain was linked with contamination of privacy curtains, bed surfaces, equipment and mop heads [5]. In 2012, there was a *Bacillus cereus* outbreak in a Singapore hospital, with the source of the infection being healthcare linen [6].

In the UK effective risk management in the infection control of hospital-associated bed linen and scrubs via in house or industrial laundering is a well-controlled process designed to minimise microbial contamination, unlike healthcare uniforms, which are often domestically laundered and may not meet the same rigorous standards.

In 2021, a study estimated that >80% of the nurses (in the UK) launder their uniform using their own domestic laundry machine (DLM) [7]. In order to perform a sufficient level of cleaning and disinfection and protect healthcare workers’ households from the transmission of infectious disease from their uniforms, four main recommendations are given by the “Uniforms and workwear: guidance for NHS employers” (2020): i) separate the uniforms from other domestic garments and textiles; ii) wash the textile at the highest temperature possible (60°C for 10min killing almost all bacteria and 30°C with detergent killing MRSA and most bacteria); iii) do not overload the machine and iv) clean the DLM regularly [8].

DLMs are preprogramed and except for the temperature and the spinning speed, users cannot control or validate the washing cycle parameters. Users rely on the manufacturer programmes without being able to check if it meets the recommendations for the thermal disinfection. In addition, little is known about the long-term performance of DLMs. Finally, variation in detergent use, addition of laundry supplements and the hardness of the water can affect the laundering performance [9].

Textiles laundered in DLMs have previously been identified as sources of infectious disease outbreaks. In 2012, postsurgical infection caused by *Gordonia bronchialis* were linked to surgical scrubs contaminated by a DLM colonised by the bacteria [10]. In a neonatal ward, an extended spectrum beta lactamase producing *Klebsiella oxytoca* isolate was transmitted to new-borns and infants through knitted clothing laundered using an onsite DLM [11].

DLMs are not a sterile environment and offer conditions favourable to the formation of biofilms [12,13]. Two major bacteria classes have been detected in DLM biofilms: Alphaproteobacteria and Gammaproteobacteria [13,14]. The presence of antibiotic resistance genes has also been detected, including efflux pumps (the three most common ones being *rsmA*, *mdsB* and *ceoB*), target modificatory (EF-TU, *rpsL* and *rpoC*) and antibiotic inhibitors genes (including AAC(6’)-lb7, *aadS*, APH(3”)-lb and various beta-lactamase encoding genes) [15,16]. Biofilms are known to facilitate the development of antimicrobial and antiseptic resistance and tolerance to detergents in DLM biofilms has been reported [12,17]. Gattlen et al (2010) observed that *Pseudomonas putida* isolated from a DLM biofilm was able to survive detergent treatment at ten times the concentration needed to eliminate the type strain [12].

Bacterial tolerance to biocides used within the environment can contribute to cross-resistance to different groups of antibiotics. Although biocides and antibiotics differ fundamentally in their chemical properties, biocides mechanisms of action are broad targeting multiple functional and structural components of microorganisms, including cell walls, membranes, nucleic acids, and proteins [18]. In contrast, antibiotics typically target specific biochemical pathways or structures, such as the inhibition of cell wall synthesis by β-lactams, Fluoroquinolones inhibit the activity of topoisomerases, or Tetracyclines which inhibits protein synthesis by binding to the ribosomal 30S subunit [19].

Despite these differences, exposure to sub-lethal concentrations of biocides can enable bacteria to develop generalized mechanisms for resistance to antibiotics. Common adaptations include the overexpression of efflux transporters and altered porin expression, while some bacteria can enzymatically degrade certain biocides, such as peroxides [19]. These generalised resistance mechanisms can also confer resistance to certain antibiotics. For example, efflux pumps—often employed to expel biocides can also enable resistance to multiple antibiotic classes, including β-lactams and fluoroquinolones. Adkin *et al.* (2022) demonstrated sub-inhibitory concentrations of biocides can lead to antibiotic resistance, even with the absence of stable biocide tolerance [20]. Another recent study highlighted a link between efflux pumps and resistance of *P. aeruginosa* to benzalkonium chloride leading to a cross resistance to cetrimide and ciprofloxacin [21]. DLMs pose a potential risk of healthcare textiles transporting domestic detergent tolerant bacteria into a healthcare environment, feeding directly into the issue of antimicrobial resistance in a clinical setting.

In conclusion, HAIs remain a critical challenge to patient safety, particularly due to the rise of antibiotic-resistant pathogens. While robust infection control measures focus on hand and surface disinfection, healthcare textiles such as uniforms represent an underexplored yet significant fomite for microbial transmission. Domestic laundering of healthcare uniforms, widely practiced by healthcare professionals in the UK and USA, raises concerns about inadequate disinfection standards and the risks posed by biofilms in DLMs. Evidence of DLM-associated outbreaks highlights the urgent need for stricter guidelines and improved validation of washing parameters. A holistic approach to infection control, incorporating both surface disinfection and the safe management of healthcare textiles, is essential to mitigate the risks posed by HAIs and protect both patients and healthcare workers. This study aims to determine the performance and ability of DLMs to decontaminate microorganisms from textiles, establish the microbiome of DLMs including the harbouring of antibiotic resistance genes and tolerance to domestic laundry detergents, in order to establish the role of DLM in the transmission of HAIs and antibiotic resistance.

## Materials and methods

### Domestic Laundry machine performance and decontamination ability

The decontamination efficacy of six separate household DLMs was assessed using *E. faecium* ATCC 6057 bioindicators (DES Controller KT4–6, Meducomp, Germany) [22]. The DES controller bioindicators used, enclose *E. faecium* contaminated swatches in a bacteria-impermeable membrane, allowing for the safe and direct testing of the DLM’s antimicrobial performance while minimising the risk of bacterial spread in a standard household setting [22]. Two bioindicators were placed in a polycotton bag and laundered at 60°C (full-length and rapid wash cycles DLMs programs) with polycotton makeweights at the DLM maximum weight capacity (Table 1). Each wash cycle was performed with either biological (14g per kilogram of fabric) or non-biological detergents (20g per wash). The temperature and duration was monitored throughout the wash cycle using a Thermochron iButton (Measurement Systems Ltd., UK) datalogger placed in the drum alongside the makeweights and the bioindicators. The log_10_ reduction of *E. faecium* was calculated using a semi-quantitative method. Briefly, laundered swatches (10^6^–10^3^ CFU swatches contained in the bioindicator) were placed in 10 ml tryptone soya broth (Oxoid, UK) and incubated at 37°C for 48 hours. An uninoculated broth was included as a control. Broths were inspected for growth prior to sub-culturing on nutrient and Slanetz and Bartley agar. Log_10_ CFU reductions were calculated based on positive growth on Slanetz and Bartley agar.

**Table 1:** Domestic Laundry machine information and thermal monitoring results.

		Machine					
		B	C	D	E	F	G
**Brand**		Hoover	Indesit	AEG	Indesit	Beko	Indesit
**Model**		HBWD8514DC-80	IW007143	7000 series kombi	IWE91281	WDEY854P44QW	IWSD61251 Eco
**Machine Age (years)**		4	2	3	9	1.4	8
**Stated Capacity (kg)**		8	7	7	9	8	6
**Actual Capacity (kg)**		5	5	4.75	2.25	4	3
**Full-Length Cycle**	**Cycle Length**	1 h 55	2 h 40 min	2 h 30	3 h 5 min	2 h 5 min	1 h 52 min
	**Peak Temperature (°C)**	57.89 ± 0.15	58.14 ± 0.20	57.14 ± 0.29	**20.48 ± 0.41**	57.76 ± 0.13	57.05 ± 0.50
	**Holding Time (±1°C; mins)**	8.00 ± 0.71	10.50 ± 0.29	9.50 ± 1.94	28.50 ± 2.75	39.25 ± 0.75	5 ± 1.15
**Rapid Cycle**	**Cycle Length**	59 min	2 h 30 min	1 h 4 min	1 h 44 min	44 min	1 h 25 min
	**Peak Temperature (°C)**	56.51 ± 0.24	57.89 ± 0.25	**44.50 ± 1.90**	**19.72 ± 0.48**	57.14 ± 0.29	**29.95 ± 1.70**
	**Holding Time (±1°C; mins)**	7.00 ± 1.08	12.75 ± 1.31	2.50 ± 0.50	20.50 ± 4.17	10.5 ± 0.96	38.25 ± 7.82

### Domestic laundry machine microbiome sampling, DNA extraction and quantification

A total of 12 DLMs were tested, six of which were previously tested for their performance and an additional six DLMs, were sampled to determine their microbiome diversity. Information about the machine (models and brands) and their usage (including number and type of cycles used, the type of detergent used and the frequency of machine cleansing) was obtained.

Two areas of each of the 12 selected DLMs were sampled: the entrance of the detergent drawer pipe and the bottom of the rubber seal of the drum (near the door). Sampling was conducted using a cotton swab moistened by PBS over a 25 cm^2^ area. After sampling, the swab head was transferred to a microcentrifuge tube and stored at +4°C for a maximum of a week or stored at -80°C for longer storage period. The DNA of the organisms collected by the swab head was extracted using the FastDNA soil spin kit (MP biomedicals, Germany) following the manufacturer’s instructions with the following modification. The swab head was transferred to the lysis matrix E tube. Then, 978 μl of sodium phosphate buffer was used to wash the microcentrifuge tube containing the swab. The sodium phosphate buffer was then transferred to the Lysis matrix tube with the swab head. The DNA extraction was then performed as instructed. The DNA concentration was determined using a Qubit™ 3.0 fluorometer (Invitrogen, UK) with the dsDNA Quantitation High Sensitivity kit (Invitrogen, UK).

Among the 24 samples (from 12 DLMs), 12 samples (from eight DLMs) which exhibited sufficient DNA concentrations (> 10ng/µl) were analysed by shotgun metagenomic sequencing (Supplementary method 1, Fig S1).

### Bacteria strain and culture

*Staphylococcus aureus* NCTC 10788, *Klebsiella pneumoniae* NCIMB 10341, and *Pseudomonas aeruginosa* ATCC 15442 were used during this study. The bacteria strains were stored at -80°C using Protect Beads (SLS, UK) until use. All bacterial strains were grown aerobically on nutrient agar (Oxoid, UK) at 37°C for 24h.

### Laundry detergent tolerance induction assay

**Fig 1:**
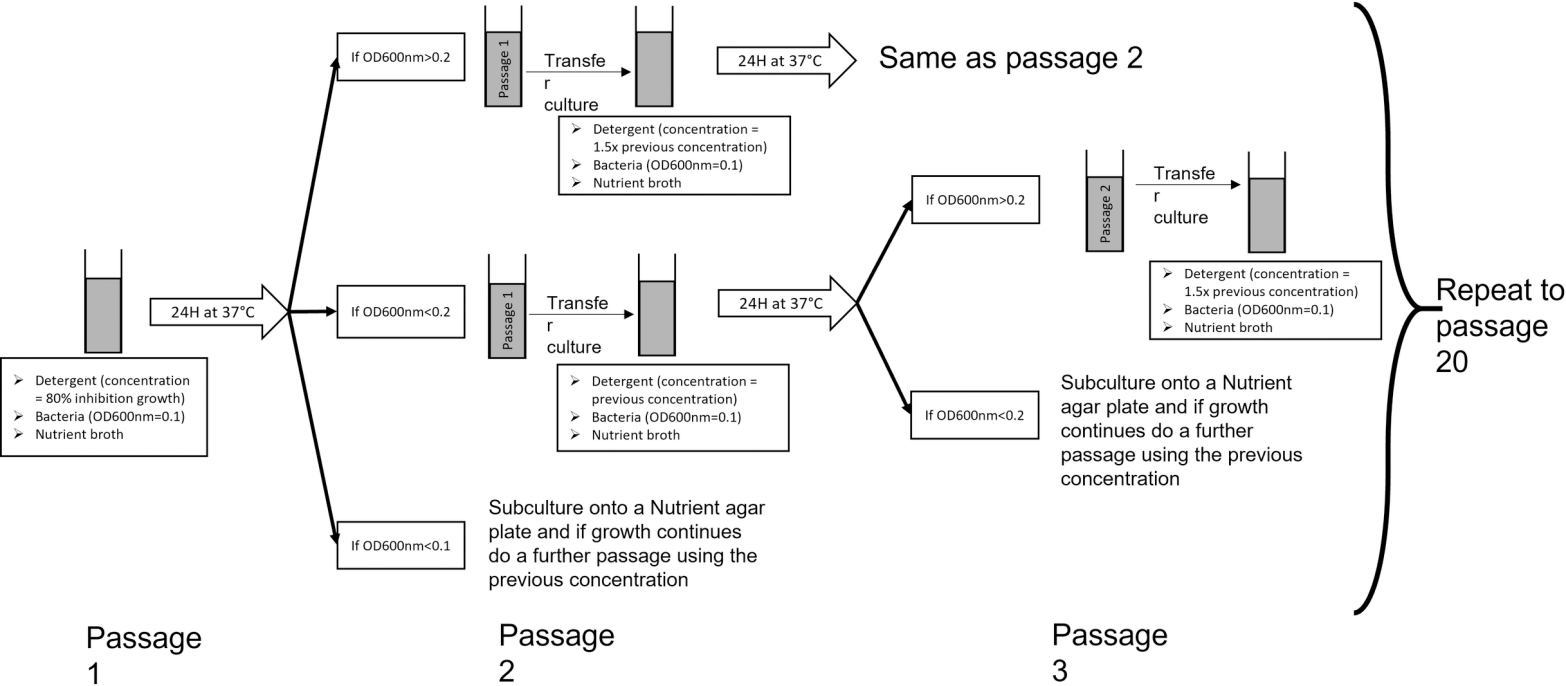
Schematic representation of the detergent tolerance induction assay.

The method used was adapted from Walsh *et al.*, (2003) [23]. In brief, a bacterial culture (final OD_600nm_ of 0.1) was prepared in nutrient broth (total volume 9ml) with the addition of 1ml of non-biological liquid detergent or non-biological powder detergent (non-biological detergent was selected rather than biological detergent as biological detergent contains enzymes and other potentially disruptive components that may influence the assay) at a concentration corresponding to 80% inhibition of growth or if 80% could not be reached the highest concentration allowing growth (Fig 1 and Table 2) (Supplementary method 2). After 24h incubation the optical density at OD_600nm_ was measured. If the optical density was greater than 0.2, the bacteria solution was diluted to an OD_600nm_ of 0.1 in a new test tube with an increased detergent concentration of x1.5. If the optical density was lower than 0.2 the bacteria solution was diluted to an OD_600nm_ of 0.1 but the same concentration of detergent was used for the next passage. The bacteria were passaged up to a maximum of 20 times. If an OD_600nm_ of below 0.1 was detected, or there were 2 consecutive readings of less than 0.2 recorded, the bacteria were sub-cultured onto a nutrient agar plate and incubated for 24 hours. If growth was present, the passages were continued; if no growth was recorded the bacterium was deemed to be unable to survive the current concentration of the detergent. Every five passages, the bacterial solution was sub-cultured onto a nutrient agar plate. The resulting growth was used for antibiotic susceptibility testing and the colonies were harvested and preserved at -80°C on Protect Beads (SLS, UK). Negative (growth media only) and positive growth controls (bacteria and growth media) were also included.

**Table 2:** Sublethal laundry detergent concentration and evolution of bacteria tolerance to laundry detergent after repeated exposure.

Bacteria	Non-Biological Detergent	Starting concentration (80% inhibition of growth)	Maximum concentration reached	% increase in concentration
***S. aureus***	Powder	180 µg/ml	270 µg/ml	50%
	Liquid	0.000675 µl/ml	0.60 µl/ml	89,000%
***K. pneumoniae***	Powder	1800 µg/ml	4100 µg/ml	128%
	Liquid	0.000675 µl/ml	10.36 µl/ml	>1.5m%
***P. aeruginosa***	Powder	1800 µg/ml*	2700 µg/ml	50%
	Liquid	0.00675 µl/ml	2.05 µl/ml	30 000%

*68% of MIC.

The original *S. aureus* NCTC 10788 strain, two DLM detergent tolerant mutant *S. aureus* strains isolated after 15 passages, the original *K. pneumoniae* NCIMB 10341 strain, and one DLM detergent tolerant mutant *K. pneumoniae* strain isolated after 15 passages in the powder detergent were analysed by whole genome sequencing (WGS) (Supplementary method 3).

### Antibiotic susceptibility test

The antibiotic susceptibility of the bacteria was assessed before the start of the laundry detergent tolerance induction assay, and then every 5 passages thereafter that the bacteria exerted detergent tolerance. The method was performed using the EUCAST disk diffusion method [24] using the antimicrobial agents M14 Ring (Ampicillin 10µg, Cephalothin 5µg, Colistin Sulphate 25µg, Gentamicin 10µg, Streptomycin 10µg, Sulphatriad 200µg, Tetracycline 25µg, Cotrimoxazole 25µg) (MAST, UK) for *K. pneumoniae* and *P. aeruginosa* and M13 Ring (Chloramphenicol 25µg, Erythromycin 5µg, Fusidic acid 10µg, Oxacillin 5µg, Novobiocin 5µg, Penicillin G 1 unit, Streptomycin 10µg and Tetracycline 25µg) (MAST, UK) for *S. aureus* or a selection of clinically relevant antibiotic discs (Table 3)

**Table 3:** Clinically relevant antibiotic screened against each bacterial strain.

	*K. pneumoniae* NCIMB 10341	*P. aeruginosa* ATCC 15442	*S. aureus* NCTC 10788
**Amikacin 30μg** *	+	+	
**Aztreonam 30μg** *	+	+	
**Cefoxitin 30μg** *			+
**Cefpoxidime 10μg** *	+		
**Ceftazidime 10μg** *	+	+	
**Ciprofloxacin 5μg ^**	+	+	+
**Ertapenem 10μg** *	+		
**Erythromycin 15μg** ^			+
**Fusidic Acid 10µg** *			+
**Linezolid 10μg** *			+
**Meropenem 10μg** *	+	+	
**Moxifloxacin 5μg** *	+		+
**Rifampicin 5μg** *			+
**Tetracycline 30μg** ^			+
**Vancomycin 30μg** ^			+

+ Antibiotic tested with the corresponding bacteria species.

* Oxoid, UK.

^ SLS, UK.

The zone of inhibition was measured and compared to the EUCAST clinical breakpoints (EUCAST, 2024) to determine the sensitivity of the organism to the antibiotic [25].

### Statistical analysis

Investigations were conducted in duplicate a minimum of two separate times. All statistical analysis was conducted using SPSS (Version 29, IBM). The normality of the data was assessed using both a Q-Q plot and the Shapiro-Wilk test for normality. The observed quantiles were compared to the theoretical normal quantiles, and p > 0.05 indicated the data did not significantly differ from normal distribution. Two-tailed t-tests were used to compare the laundry detergent tolerance induction assay pre-treatment zones of inhibition and post-treatment zones of inhibition. Values of p≤0.05 were considered to be statistically significant.

## Results

### Decontamination efficacy of domestic laundry machines

Five out of the six washing machines tested at 60°C for a full-length wash had a peak temperature ranging from 57.05°C -58.14°C, held for between 5–39 minutes (Table 1 and Fig S2). A ≥5 log_10_ CFU reduction was achieved by four of the five machines using full-length cycles (Table 4). Machine E reached a mean peak temperature of 20.48°C for 28.5 minutes and did not result in an observable reduction in *E. faecium* (Tables 1 and 4).

**Table 4:** Log_10_ reduction of *E. faecium* bioindicators following domestic laundering using a 60°C full-length or rapid wash cycle (n=4).

Machine	Full-Length 60°C		Rapid 60°C	
	Biological	Non-Biological	Biological	Non-Biological
**B**	0	6	≥4	6
**C**	6	6	6	6
**D**	6	6	0	0
**E**	0	0	0	0
**F**	≥5	5	5	6
**G**	0	0	0	0

Green – pass (≥5 log_10_ reduction); Red – fail (<5 log_10_ reduction).

Of the rapid wash cycles, 50% of the six machines did not reach the stated 60°C ±4°C (measured temperature, 19.72°C-44.5°C), and failed to decontaminate *E. faecium* (<5 log_10_ CFU reduction; Tables 1 and 4). Conversely, two machines reached a peak temperature of 56.51°C-57.89°C for 7–13 minutes and achieved a ≥5 log_10_ CFU reduction. One machine (B) achieved a 6 log_10_ CFU reduction when decontaminated using a rapid cycle with a non-biological detergent. However, its decontamination efficacy was variable when using a biological detergent, achieving a reduction of ≥4 log_10_ CFU.

### Domestic machine microbiome and resistome

After DNA extraction, only 12 samples (from eight different DLM) reached a DNA concentration greater than 10ng/μl. Those 12 samples were further analysed by shotgun metagenomic sequencing (Table 5). Eight samples were pairs (detergent pipe and door rubber) from four DLM (pairs A1/A2, E1/E2, H1/H2 and M1/M2) and four samples were single samples from four different DLM (C1, F2, L1 and O1). The usage survey results are summarised in Table 5.

**Table 5:** Results survey domestic machine usage and DNA concentration.

Sample Name	Sampling zone	DNA concentration (ng/μl)	Machine code	Detergent type used	Use of Softener	Detergent addition location	At least one wash >50°C per month	Machine age^†^	Use machine cleanser
**A1#**	Pipe	**22.6**	A	Non-biological	No softener	Drum	No	Old	No
**A2#**	Seal	**190**							
B1	Pipe	0.19	B	Non-biological	No softener	Compartment	Yes	New	Yes
B2	Seal	LLD							
**C1#**	Pipe	**76.8**	C	Non-biological	Softener	Compartment	No	New	No
C2	Seal	1.08							
D1	Pipe	2.68	D	Non-biological	Softener	Drum	Yes	New	No
D2	Seal	2.94							
**E1#**	Pipe	**20.2**	E	Biological	No softener	Drum	No	Old	No
**E2#**	Seal	**68.2**							
F1	Pipe	0.924	F	Non-biological	No softener	Compartment	No	New	Yes
**F2#**	Seal	**21.8**							
G1^*^	Pipe	1	G	N/A	No softener	N/A	N/A	Old	Yes
G2^*^	Seal	4.32							
**H1#**	Pipe	**57**	H	Biological	Softener	Compartment	No	Old	Yes
**H2#**	Seal	**114**							
**L1#**	Pipe	**92**	L	Non-biological	Softener	Drum	Yes	Old	No
L2	Seal	5.68							
**M1#**	Pipe	**21.2**	M	Biological	Softener	Drum	No	Old	Yes
**M2#**	Seal	**22.8**							
N1	Pipe	1.99	N	Non-biological	Softener	Drum	Yes	New	No
N2	Seal	1.68							
**O1#**	Pipe	**46.4**	O	Non-biological	No softener	Drum	Yes	New	No
O2	Seal	LLD							

LLD= lower than the limit of detection

* DLM located in a research laboratory used for research purpose only.

† Old >4 years; New ≤ 4 years.# Samples used for the shotgun metagenomic sequencing

N/A: non-applicable as the usage conditions are extremely variable due to DLM research purpose.

**Fig 2:**
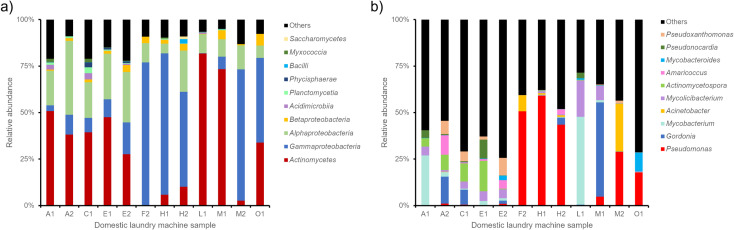
Relative abundance of the ten most common classes and genera among the samples. **a)** Relative abundance of microbial classes **b)** Relative abundance of bacteria genera.

The microbiome analysis of the samples showed that three main classes of bacteria represented >60% of the bacteria detected in all the samples: Actinomycetes, Gammaproteobacteria and Alphaproteobacteria (Fig 2a). The ten most common genera among the samples were *Pseudomonas, Mycobacterium, Gordonia, Acinetobacter, Mycolicibacterium, Actinomycetospora, Amaricocus, Mycobacteroides, Pseudonocardia* and *Pseudoxanthomonas*(Fig 2b). Three sample pairs (A1/A2, E1/E2 and M1/M2) showed different microbiome profiles between the detergent pipe sample and the drum rubber sample even though they were sampled from the same machine. The major genus detected in sample A1 was *Mycobacterium* and for A2 it was *Gordonia*. For DLM E the major genus detected was *Actinomycetospora* in the detergent pipe (E1) and *Pseudoxanthomonas* in the drum rubber sample (E2). The samples M1 and M2 exhibited as major genera *Gordonia* and P*seudomonas* respectively. One machine (H) exhibited the same major genus: *Pseudomonas* (Fig 2b) within the DLM sample pair. In all the samples, the presence of genera containing known pathogenic bacteria species was detected (Table 6). Three genera showed a relative abundance greater than 1% of the total bacteria*: Acinetobacter* (9–28%), *Mycobacterium* (1–49%) and *Pseudomonas* (1–61%). For 11 samples, at least one of the three genera above represented more than 1% of the detected bacteria and for eight samples at least one represented more than 10% of the detected bacteria (Table 6).

**Table 6:** Relative abundance of potentially pathogenic genera detected in domestic laundry machine microbiome. Light grey is genera with relative abundance ≥ 1%, genera with a relative abundance ≥ 10% highlighted in dark grey.

	Percentage of total bacteria detected in each sample^a^											
	A1	A2	C1	E1	E2	F2	H1	H2	L1	M1	M2	O1
*Acinetobacter*	0.002	0.2	0.009	0.007	0.05	**9**	0.9	0.9	0.001	0.004	**28**	0.03
*Bacillus*	0.007	0.005	0.006	0.007	0.009	0.009	0.002	0.4	0.0004	0.01	0.002	0.00002
*Citrobacter*	0.002	0.0001	0.0002	0.0005	0.0003	0.0004	0.001	0.0003	0.00003	0.0009	0.001	0.0002
***Clostridioides***	0.000003	0.02	0.02	0.0005	0.006	0.00001	0.00001	0.001	0.0002	0.0003	ND	0.00001
Enterobacter*	0.0003	0.0005	0.002	0.001	0.0006	0.01	0.2	0.002	0.0007	0.002	0.2	0.008
Enterococcus*	0.001	0.0003	0.001	0.001	0.001	0.0005	0.0002	0.0003	0.0004	0.0008	0.0009	ND
*Escherichia*	0.002	0.001	0.004	0.002	0.001	0.009	0.005	0.008	0.002	0.001	0.009	0.005
Klebsiella*	0.001	0.002	0.005	0.001	0.003	0.02	0.03	0.02	0.0005	0.001	0.02	0.01
*Mycobacterium*	**29**	**3**	0.6	**2**	**1**	0.001	0.3	0.4	**49**	**1**	0.002	0.2
Pseudomonas*	0.06	**1**	0.5	0.1	**1**	**52**	**61**	**46**	0.05	**5**	**31**	**18**
Staphylococcus*	0.004	0.0001	0.0008	0.0007	0.0007	0.0001	0.0004	0.006	0.0004	0.0002	0.001	0.003
*Streptococcus*	0.0007	0.0004	0.0003	0.0001	0.0006	0.001	0.003	0.002	ND	0.0001	0.002	0.0008

^a^Each letter and digit combination correspond to one DLM and sample site.

ND= Not detected

*Genera containing species belonging to the ESKAPE pathogens

**Fig 3:**
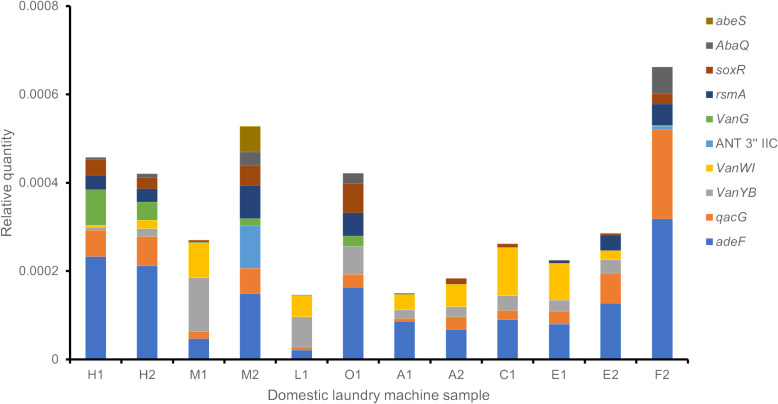
Abundance of resistance genes in each DLM samples. The 10 most abundant resistance genes in the samples are displayed.

Antibiotic resistance genes could be detected in all the samples sequenced. The ten most common genes are listed in Table 7 and Fig 3. Five genes encode for efflux pumps (*adeF, qacG, rsmA, abaQ* and *abeS*), three genes encode for altered variants of the antibiotic targets (*vanYB*, *vanWI* and *vanG*), one gene encodes an antibiotic inhibitor (ANT 3” IIC) and one gene is involved in the expression of efflux pumps and genes altering the antibiotic targets (*soxR*) (Table 7).

**Table 7:** Antibiotic resistance genes detected in domestic laundry machines. The resistance genes are ranked from the highest frequency (top) to the lowest frequency (bottom) among all the samples. Only the 10 most frequent ones are listed. Gene data were extracted from the CARD database. Blue= Genes encoding efflux pumps; Yellow=Genes encoding an antibiotic inhibitor; Green= Genes involved in antibiotic targets alteration; and Grey=Genes involved in different antibiotic.

Gene	Resistance mechanism	Drug class
***adeF***	Antibiotic efflux	Tetracycline antibiotic, fluoroquinolone antibiotic
***qacG***	Antibiotic efflux	Disinfecting agents and antiseptics
***vanYB***	Antibiotic target alteration	Glycopeptide antibiotic
***vanWI***	Antibiotic target alteration	Glycopeptide antibiotic
***ANT 3’‘ IIC***	Antibiotic inactivation	Aminoglycoside antibiotic
***vanG***	Antibiotic target alteration	Glycopeptide antibiotic
***rsmA***	Antibiotic efflux	Diaminopyrimidine antibiotic, phenicol antibiotic, fluoroquinolone antibiotic
***soxR***	Antibiotic efflux and Antibiotic target alteration	Tetracycline antibiotic, cephalosporin, penam, fluoroquinolone antibiotic, phenicol antibiotic, disinfecting agents and antiseptics, rifamycin antibiotic, glycylcycline
***abaQ***	Antibiotic efflux	Fluoroquinolone antibiotic
***abeS***	Antibiotic efflux	Macrolide antibiotic, aminocoumarin antibiotic

### Sublethal concentrations of domestic laundry detergent

Sublethal concentrations (see Supplementary method 2) (80% growth inhibition) for *S. aureus* were 0.000675 µl/ml for the liquid detergent, and 180 µg/ml for powder detergent. For *K. pneumoniae* 0.000675 µl/ml was required for the liquid detergent, and 1800 µg/ml for the powder detergent. The sublethal concentrations for *P. aeruginosa* were 0.00675 µl/ml for liquid detergent, for the powder detergent only a 68% inhibition was achievable at 1800 µg/ml. Growth inhibition couldn’t be achieved at 80% of *P. aeruginosa*, therefore, 68% inhibition of growth was used for the induction assay.

### Laundry detergent tolerance and cross resistance to antibiotics

During the laundry detergent tolerance induction assay, the detergent concentration was increased by at least one stable (1.5x) increase of the sublethal starting concentration for all bacterial strains and detergent types tested. The powdered non-biological detergent exhibited the lowest number of stable increases of the sublethal starting concentration for all the bacteria strains (Table 2). *S. aureus* increased from 180 µg/ml to 270 µg/ml (a 50% increase), *K. pneumoniae* increased from 1800 µg/ml to 4100 µg/ml (a 128% increase), and *P. aeruginosa* increased from 1800 µg/ml to 2700 µg/ml (a 50% increase). Whilst liquid laundry detergent consistently demonstrated a higher number of stable increases of the sublethal starting concentration (Table 2). *S. aureus* increased from 0.000675 µl/ml to 0.60 µl/ml (an 89,000% increase), *K. pneumoniae* increased from 0.000675 µl/ml to 10.36 µl/ml (a>1.5m% increase), and *P. aeruginosa* from 0.00675 µl/ml to 2.05 µl/ml (a 30,000% increase) (Table 2).

**Fig 4:**
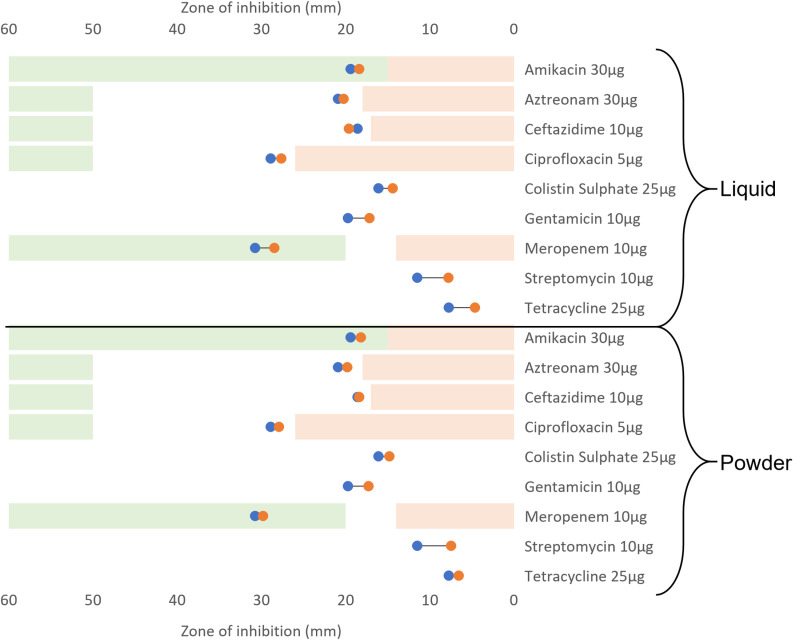
*P. aeruginosa* antibiotic resistance profile before and after repeated exposure to laundry detergents (liquid or powdered). Blue markers represent the mean ZoI before exposure to the detergents. Orange markers represent the mean ZoI after exposure to the detergents. The green areas indicate each antibiotic susceptibility area based on the EUCAST (2024) breakpoints [25], the white areas represent the intermediary susceptibility, and the light red areas represent the antibiotic resistance area. When the antibiotic is not clinically relevant no areas is highlighted.

Both *S. aureus* and *K. pneumoniae* were screened against eight antibiotics as part of the M13/14 Rings. Only four of these were considered for *P. aeruginosa* in the M14 ring due to Ampicillin 10µg, Cephalothin 5µg, Sulphatriad 200µg, and Cotrimoxazole 25µg being ineffective against *Pseudomonas*. After 20 passages in both liquid and powder detergent, all antibiotics tested against *P. aeruginosa* demonstrated a decrease in zone of inhibition (ZoI), except for Ceftazidime 10µg (a 1mm increase in ZoI). The greatest difference observed pre and post exposure to detergent was for Streptomycin with 3.71mm and 4mm ZoI reduction for the liquid and the powder detergents respectively (Fig 4, Table S1). Based on the EUCAST sensitivity breakpoint, *P. aeruginosa* is clinically susceptible to the antibiotics tested before and after exposure to laundry detergents. *P. aeruginosa* was fully resistant to four of the eight M14 ring antibiotics (Ampicillin 10µg, Cephalothin 5µg, Sulphatriad 200µg, and Cotrimoxazole 25µg) prior the treatment with the detergents and remained resistant after repeated exposure to the detergents. In *P. aeruginosa* a significant correlation between detergent exposure/concentration and decrease in ZoI against antibiotics was observed (p ≤ 0.05) independently of the detergent form (powder or liquid).

**Fig 5:**
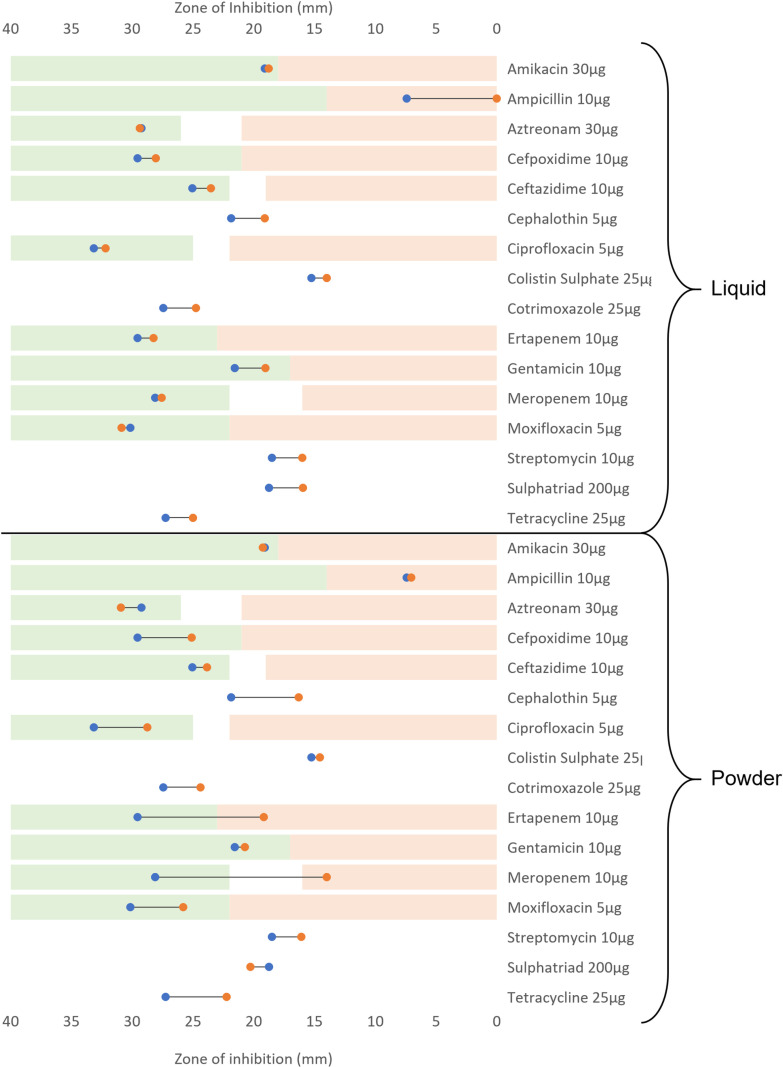
*K. pneumoniae* antibiotic resistance profile before and after repeated exposure to laundry detergents (liquid or powdered). Blue markers represent the mean ZoI before exposure to the detergents. Orange markers represent the mean ZoI after exposure to the detergents. The green areas indicate each antibiotic susceptibility area based on the EUCAST (2024) breakpoints [25], the white areas represent the intermediary susceptibility, and the light red areas represent the antibiotic resistance area. When the antibiotic is not clinically relevant no areas is highlighted.

There is a significant correlation between detergent exposure and the changes in antibiotic ZoI observed (p ≤ 0.05) in *K. pneumoniae*. The largest change in the ZoI seen against *Klebsiella pneumoniae* was for Meropenem 10µg, which had a difference of 14.12mm when exposed to domestic non-biological powder (Fig 5, Table S2). When exposed to liquid detergent the largest change was observed with Ampicillin 10µg (-7.41mm). Two instances of the induction of cross-resistance to antibiotics were observed following powder detergent exposure: Meropenem 10µg, and Ertapenem 10µg. Following exposure to powder and liquid detergents, the general trend observed was an increase in ZoI when antibiotics were tested against *K. pneumoniae*. There were five occurrences of an increased ZoI when antibiotics (Moxifloxacin 5μg (liquid), Aztreonam 30 μg (both), Amikacin 30 μg (powder), and Sulphatriad 200 μg (powder)) were tested against *K. pneumoniae*.

**Fig 6:**
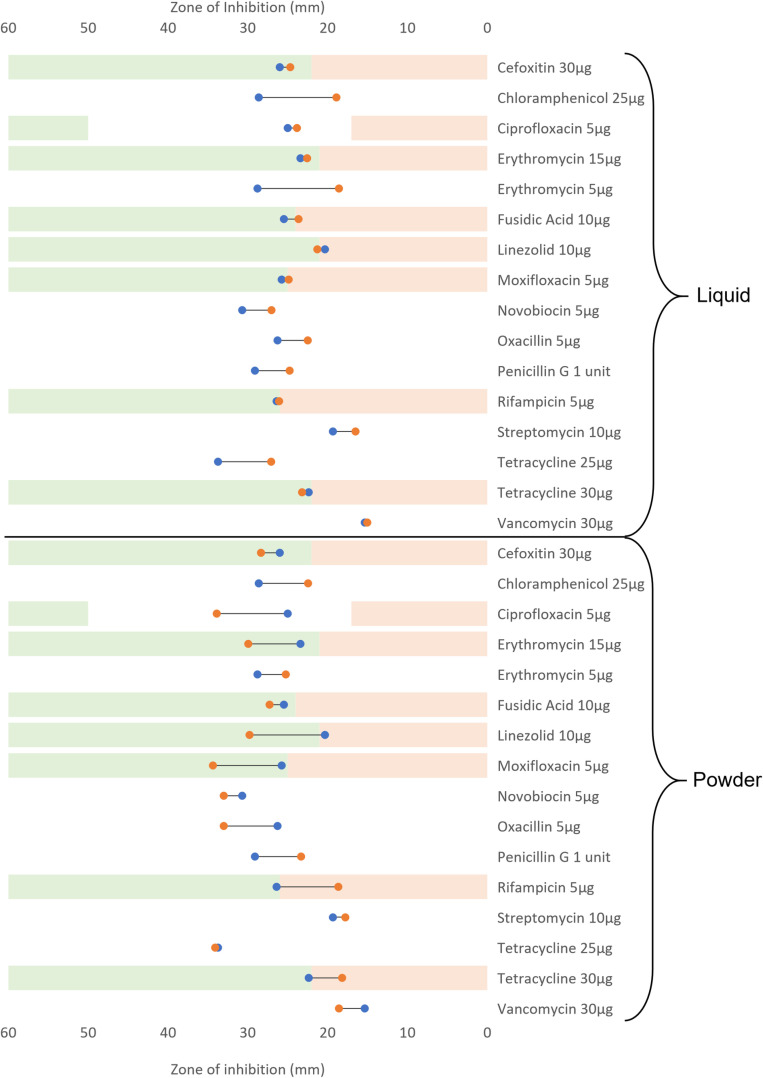
*S. aureus* antibiotic resistance profile before and after repeated exposure to laundry detergents (liquid or powdered). Blue markers represent the mean ZoI before exposure to the detergents. Orange markers represent the mean ZoI after exposure to the detergents. The green areas indicate each antibiotic susceptibility area based on the EUCAST (2024) breakpoints [25], the white areas represent the intermediary susceptibility, and the light red areas represent the antibiotic resistance area. When the antibiotic is not clinically relevant no areas is highlighted.

*S. aureus* had variable changes to sensitivity in both directions to the antibiotics after exposure to DLM detergent. Rifampicin 5µg screening demonstrated a 7.73mm decrease in ZoI, while the largest increase in ZoI was against linezolid 10µg, which increased by 9.43mm (Fig 6, Table S3). A significant relationship was observed between domestic liquid detergent exposure and a decrease in the ZoI observed in *S. aureus* (p ≤0.05). However, there was no significant relationship identified between the powder detergent exposure, and the resistance profiles in the post treatment strain (p≥0.05).

Across all the bacterial species tested, there were nine instances of cross-resistance to antibiotics from exposure to domestic laundry detergents. *K. pneumoniae* exposure to powder detergent induced cross resistance to Ertapenem 10μg (a ZOI reduction of 10.38mm) and Meropenem 10μg (a ZOI reduction of 14.12mm). *S. aureus* assays demonstrated, seven antibiotic resistances. *S. aureus* exposed to liquid detergent became resistant to Moxifloxacin 5μg (a 0.9mm ZOI reduction), Fusidic Acid 10μg (a ZOI reduction of 1.83mm), and Penicillin G 1 Unit (a 4.32mm ZOI reduction). *S. aureus* exposed to powder detergents then demonstrated resistance to Tetracycline 3μg (a ZOI reduction of 4.21mm), Rifampicin 5 μg (a 7.73mm reduction of the ZOI), Penicillin G 1 unit (a reduction of 5.75mm to the ZOI), and Oxacillin 5μg (a ZOI reduction of 6.72mm).

### Whole Genome Sequencing of antibiotic cross-resistant strains

The WGS analysis showed the presence of a single nucleotide polymorphism (SNP) in the genome of *S. aureus* following exposure to the liquid detergent. This SNP was detected in the coding region of the *Mgra* gene and induced a non-synonymous missense mutation substituting an arginine by histidine in the amino acid sequence. Two different SNPs were identified in *K. pneumoniae* genome following exposure to detergent powder. The first SNP was detected in the *AcrB* gene, encoding a multidrug efflux RND (Resistance Nodulation-Division) transporter permease. The presence of the SNP in the post treatment isolate induces the substitution of a stop codon with a tryptophan in the AcrB protein. The second SNP is located in the *murB* gene and induces a silent mutation and thus no amino acid change occur in the protein sequence.

## Discussion

The performance of domestic laundry machines (DLMs) in achieving effective disinfection presents significant challenges, particularly in the context of healthcare worker uniforms, which could act as fomites for HAIs. Variability in DLM cycle parameters, such as temperature and duration, raises concerns about their reliability in reaching the disinfection standards necessary to eliminate pathogenic microorganisms. This issue is compounded by the limited efficacy of domestic detergents when used below the concentration required to kill microorganisms and the potential for biofilm formation within DLMs, creating an environment conducive to antimicrobial resistance. Understanding these risks and their implications is critical to improving laundering practices, safeguarding healthcare environments, and addressing the broader challenge of infection control.

The performance of DLMs varied between machines, with an important variation in cycle lengths and temperature profiles. Of particular significance is that all DLMs did not reach the target temperature of 60°C during a rapid wash cycle and 50% (3/6) and 33% (2/6) failed to achieve disinfection (Tables 1 and 4) when using a short wash cycle and standard wash cycle respectively. Only two of the DLMs could maintain their peak temperature >57°C for the Department of Health recommended 10 minutes, although the temperature maintained was below that of the 60°C recommended [8]. This suggests that even if healthcare workers thought they were disinfecting their uniforms at 60°C for 10 minutes there is a high possibility that the machine used will not perform as expected. During this study, three DLMs (50%) did not reach temperature (60°C ±4°C) and maintain it for 10 minutes and disinfection was not achieved despite the presence of detergent (Table 4). It is however difficult to determine the antimicrobial efficacy of the detergent itself from this study investigations. Several studies have showed that the HAI organisms MRSA and *A. baumanii* and other Gram-negative bacteria can survive washes performed under 60°C without detergent [26,27]. If the temperature to disinfect textiles (60°C) can’t be reached then importance should be given to the laundry detergent. Standard domestic detergents are not necessarily designed for disinfection and may not be able to kill bacteria so priority should be given to detergents with proven antimicrobial properties. In addition, the detergents should be used at a concentration high enough to kill the microorganisms. Firstly, to have an efficient killing of the potential pathogen present on the textile but also because concentrations below that required to kill bacteria may lead to the development of detergent tolerance and cross resistance to antibiotics. Controlling the concentration of detergents used in a DLM can be challenging. Indeed, the water volume used by the DLM for the wash cycles are generally unknown and can vary with the wash load. In addition, the quality of the water may affect the efficiency of the domestic detergent. Water hardness has been shown to affect detergent and disinfectant efficacy. Linear alkylbenzene sulfonate is less effective at removing soiling when dissolved in hard water (around 50% and 20% removal on cotton towels and cotton woven textiles respectively) compared to deionised water (around 80% and 40% removal respectively) [9]. The antimicrobial activity of some disinfectant can also be negatively affected by the water hardness, whereby sodium hypochlorite achieves a lower level of disinfection against *E. coli* and SARS-CoV-2 when in hard water [28,29]. Detergent manufacturers give instructions based on washing load, soiling level and water hardness but those instruction can be difficult to understand or follow when the individual doesn’t know the DLM features and the water quality in their area.

For HCW, an underperforming DLM is an important issue when considering uniform laundering. Indeed, there are several examples of the role of textile, in particular garments, in the spreading of pathogenic bacteria in healthcare settings. In 2012, an anaesthetic nurse’s scrubs were identified as responsible of spreading *G. bronchialis* to 3 patients [10]. Although, the nurse’s DLM couldn’t be tested for the presence of the bacteria, the bacteria strain was found on the nurse’s body and scrubs and the outbreak stopped after the disposal of their washing machine. More recently, in 2019, a β-lactamase producing *Klebsiella oxytoca* strain spread through a paediatric ward with 13 new-borns and one child testing positive [11]. The bacterium was detected inside the DLM used in the ward to wash the newborn hats and socks, as well as being detected in two sinks. As with the *G. bronchialis* case, the spread of the bacteria was stopped by removing the contaminated washing machine.

Pathogenic bacteria transmission can originate from healthcare settings, however, DLMs can also be a potential source of contamination due to them having their own microbiome. Among the 12 DLMs selected for this study, four machines could not have their microbiome and resistome analysed due to insufficient DNA concentration in the samples (Table 5). Interestingly, all excluded samples were from DLMs less than 4 years old. Since the volume of DNA extracted is likely associated with the amount of biofilm present at the sampling sites, the observed difference in DNA yield between newer and older machines could be attributed to biofilm accumulation over time, particularly in areas such as the detergent drawer pipe.

Three classes represented >60% of the total bacteria detected in this study: Alphaproteobacteria, Gammaproteobacteria and Actinomycetes (Fig 2). This is consistent with previous observations. Nix et al (2015) analysed the microbiome of 12 DLMs and observed that the Alphaproteobacteria represented in average 35% of the bacteria detected followed by the Gammaproteobacteria (30%) [13]. Similarly, Jacksch et al (2019) observed that the Alphaproteobacteria and the Gammaproteobacteria classes were the most abundant with 17.5% and 57.8% respectively [14]. A more recent study by Chen et al (2024) which analysed dormitory washing machine filters, found that Proteobacteria were the major bacteria detected in the machine filters with an average abundance of 74.73% [15]. The authors also explored the antibiotic resistance gene content of the washing machines and they observed that all the machines were positive for the presence of antibiotic resistance genes with the highest abundance observed for efflux pumps and target alteration genes. An earlier study (2020) looking for specific antibiotic resistance genes (Beta-lactamases) in 54 DLMs detected the presence of antibiotic resistance genes in 66.6% of the DLMs [16]. In accordance with previous published data, the presence of antibiotic resistance genes was also detected in all the DLMs tested during this study with a higher abundance of efflux pumps and target modification genes. Interestingly, the presence of potentially pathogenic genera was also detected in the DLMs microbiome especially *Mycobacterium*, *Pseudomonas* and *Acinetobacter* genera. The combined presence of potentially pathogenic bacteria and antibiotic resistance genes in DLMs raises the question of the potential risk that underperforming DLMs represents for the laundering of HCW uniforms in domestic settings. The misuse of domestic laundry detergents could also be a potential source of the transmission of antimicrobial resistance.

Indeed, repeated exposure to stable increases of non-biological domestic laundry detergents led to the induction of antibiotic cross-resistance in both *S. aureus* and *K. pneumoniae*. Sub-lethal concentrations of powder detergent led to the resistance of *K. pneumoniae* to both carbapenem antibiotics tested: Ertapenem 10μg (a ZoI reduction of 10.38mm) and Meropenem 10μg (a ZoI reduction of 14.13mm) (Fig 5). This combination of resistances suggests *K. pneumoniae* has become a Carbapenem-Resistant Enterobacteriaceae (CRE). To be considered a CRE, the Enterobacteriaceae must be not susceptible to at least one carbapenem antibiotic [30]. This is of concern as carbapenems are often used as last line clinical options for treatment [31]. Antibiotic cross-resistance was induced irrespective of which detergent was used in *S. aureus*. The liquid detergent assay led to the induction of resistance towards Moxifloxacin 5μg, Fusidic Acid 10μg, and Penicillin G 1 unit. Whilst resistance was generated in the powder detergent assay to Tetracycline 30μg, Rifampicin 5μg, Penicillin G 1 unit, and Oxacillin 5μg (Fig 6). These new resistance patterns suggest the original methicillin-sensitive *S. aureus* strain has become a methicillin-resistant *S. aureus* (MRSA) strain following exposure to both liquid and powder detergent; this is inferred by the induction of β-lactam resistance (Penicillin G 1 unit 29.08mm-24.76mm and 29.08mm-23.33mm, Oxacillin 5µg 26.27mm-19.55mm) [32]. Despite methicillin not being tested, oxacillin is used as an alternative for antibiotic susceptibility testing [33]. Exposure to both detergents also led to the loss of resistance of *S. aureus* towards the Oxazolidinone Linezolid 10μg (a ZoI increase of 0.93mm in liquid detergent, and 9.43mm in powder detergent). This antibiotic is used against various Gram-positive bacteria including MRSA, particularly in cases of bacteraemia, necrotizing pneumonia, joint/bone infections, and skin infections [34,35]. The induction of antibiotic resistances in the tested microorganisms is supported by previous studies investigating links between biocide tolerance and cross resistance to antibiotics. Henly *et al.,* (2019) identified six occurrences of cross-resistance to antibiotics when assessing the long-term effects of benzalkonium chloride and triclosan against *E. coli* [36]. Similarly, Kampf (2018) was able to induce cross-resistance to antibiotics in Gram-negative species after exposure to 13 different biocides [37]. In another study 43% of the strains tested showed a decrease in susceptibility to antibiotics after exposure to biocides such as glutaraldehyde, chlorhexidine, and benzalkonium chloride, along with increased capacity for biofilm formation in those antibiotic-resistant strains [38].

The ability of bacteria to tolerate increasing concentrations of detergents showed variation depending on whether they were exposed to powder or liquid detergents. This is evident in Table 2 where the percentage increase in concentration was 50% (*S. aureus*), 128% (*K. pneumoniae*), and 50% (*P. aeruginosa*) when exposed to powder detergent; liquid detergent however, demonstrated increases in concentration of 89,000% against *S. aureus*,>1.5m% against *K. pneumoniae*, and 30,000% against *P. aeruginosa*. The stated concentration increases are impacted by the number of exposures that took place. Both *S. aureus* assays stopped at 15 exposures. This is comparable to *K. pneumoniae* when exposed to powder detergent (15 exposures), but not to *P. aeruginosa*, which had 20 exposures. This was due to *P. aeruginosa* surviving for a longer period against the presence of the powder detergent. Previous studies have identified a difference in antimicrobial efficacy between detergents that contain activated oxygen bleach (AOB), and those that do not [39]. When washing at 30^o^C, a log_10_ reduction of less than 1 was observed in liquid detergent not containing AOB, compared to a log_10_ reduction of 6.3 (with a pre-wash) and 3.1 (without pre-wash) using detergent pearls containing AOB. Similarly, a difference of 4 log_10_ (pre-wash) and 2 log_10_ (without pre-wash) was identified at 60^o^C between the two detergents [40].

Exposure to domestic detergent powder leads to varying effects on antibiotic tolerance and resistance. In *K. pneumoniae* most antibiotics exhibited a reduction in ZoI, but increases were noted in Aztreonam 30µg, Amikacin 30µg, and sulphatriad 200µg. No increases in ZoI were present in *P. aeruginosa* following powder detergent exposure. The opposite effect was observed for *S. aureus*; where there were 10 instances of a ZoI increase and just 5 decreases in ZoI (4 of which were the induction of antibiotic cross-resistance).

WGS of *S. aureus* uncovered a single nucleotide polymorphism (SNP) present within the *MgrA* gene following exposure to domestic liquid detergent. A change of the MgrA protein sequence is induced, changing arginine to histidine, which may impact its function. *MgrA* is a major global regulator of *S. aureus*, it has been linked to increased virulence and antibiotic resistance as it regulates efflux pumps, including *NorA* which is involved in quinolone resistance [41,42]. *K. pneumoniae* sequencing led to the identification of an SNP located within the *AcrB* gene after powder detergent exposure. This SNP substitute a stop codon found within the pre-exposure *K. pneumoniae* with a tryptophan. The presence of a stop codon in the protein sequence shortened the length of the protein by introducing an early termination of the translation. The presence of this stop codon in the isolates that were not exposed to detergents likely means that the protein was non-functional. The substitution of the stop codon with a tryptophan may lead to the recovery of the protein function. The *AcrB* gene encodes for a subunit of the AcrAB efflux pump which has been previously identified as being involved in resistance to a range of antibiotics including β-lactams, aminoglycosides, and quinolones [43,44]. The recovery of the AcrAB efflux pump function provides a possible explanation for the increased tolerance observed for Moxifloxacin (ZoI -4.3mm) and Ciprofloxain (ZoI -4.4mm).

Further investigation on the functional impact of the mutations detected in *S. aureus* and *K. pneumoniae* genomes after exposure to the detergent are required to clarify the role of the mutations in detergent tolerance and antibiotic resistance.

The upregulation of efflux pumps could be a driver in the cross-resistances observed in *S. aureus* and *K. pneumoniae*. Quaternary ammonium compounds (QACs) are one major component found commonly in laundry detergents [22]. Studies have linked QACs to the development of cross-resistance in *S. aureus* via the upregulation of efflux pump genes, notably *NorA*. A change in regulation of *NorA* would influence *S. aureus* susceptibility to antibiotics as this efflux pump confers resistance to fluoroquinolones, β-lactams, tetracyclines, chloramphenicol, and macrolides [45]. Interestingly, increased tolerance to tetracycline and Rifampicin were observed in *S. aureus* after exposure to the powder detergent which could be linked to upregulation of the *NorA* gene (Fig 5, Table S3). The genetic modification observed in *S. aureus* and *K. pneumoniae* can only partially explain the increased tolerance to antibiotics observed during the induction assay. Gene expression investigations are required to give more insight into the potential role of the upregulation/downregulation in the antibiotic tolerance variation.

An important point to consider is that DLMs are developed and marketed to achieve an aesthetic cleaning of domestic laundry. The machines are not specifically designed to achieve microbiological disinfection. Similarly, domestic detergents if not specifically marketed as antimicrobial are designed to cleanse domestic textile items while preserving as much as possible the textile integrity rather than to achieve disinfection. The combination of the many different DLMs on the market, with the many different detergents, and the variety of machine programmes that can be selected by the consumer creates uncertainty in the level of disinfection that can be achieved using DLMs (0 log_10_–6 log_10_ reductions in the relatively small number of combinations used in this paper).

Only DLMs from standard households, not HCW-associated homes, were tested. HCW household DLMs may exhibit different microbiome and resistome profiles, with the possibility of HCW uniforms introducing greater antibiotic-resistant organisms into the DLMs from the healthcare setting. The tested DLMs’ poor performance and microbiome profile is a strong indicator that similar will be occurring in those households where HCWs reside. Further research should explore microbial exchange between garments and DLMs, including genetic transfer, to better assess the risks of DLM failure in decontaminating healthcare uniforms.

The inability of domestic laundering machines (DLMs) and household detergents to effectively disinfect healthcare workers’ uniforms poses significant infection control challenges and raises concerns about the potential emergence of antimicrobial resistance. Healthcare workers’ uniforms can serve as fomites for pathogenic microorganisms, transferring them from hospital environments to DLMs. If these microorganisms are not effectively eradicated during laundering, they can contaminate the DLM, spread to other garments, and potentially return to healthcare settings, exposing patients to harmful pathogens and increasing the risk of infection.

Moreover, the interaction between the DLM microbiome and bacterial tolerance to domestic detergents may facilitate cross-resistance or genetic transfer, further contributing to the development of antimicrobial resistance. These risks are exacerbated by findings that many DLMs fail to achieve the recommended temperatures for disinfecting healthcare uniforms, combined with the misuse or poor quality of domestic detergents.

To mitigate these risks, current guidelines for laundering healthcare worker uniforms should be revised. Key recommendations are outlined in Table 8 including; regularly cleaning DLMs to reduce biofilm accumulation and microbial contamination, using detergents with proven disinfection efficacy, providing HCW with a list of approved detergents or directly supply detergents/disinfectants with antimicrobial efficacy and prioritising onsite or industrial laundering of healthcare uniforms, where regular monitoring and maintenance of laundering systems can ensure optimal disinfection. It is important to note that even if recommendations for domestic laundering of HCW uniforms are implemented, there remains a significant risk that household machines may not reach the required temperature for effective microorganism decontamination. Preference should be given to on-site or industrial laundering, where professionally installed commercial equipment ensures controlled, monitored, and regulated wash parameters. Additionally, industrial laundries adhering to BS EN 14065:2016 standards provide controlled decontamination, ultimately improving the laundering process and reducing the risk of infectious disease transmission [46].

**Table 8:** Recommendations for the laundering of the healthcare workers uniforms.

Healthcare workers		Healthcare workers employer	
Recommendations	Reasons	Recommendations	Reasons
Do not use short cycles	Short cycles are more likely to underperform	Establish a list of domestic detergents/supply detergents, that are proven to provide chemical disinfection at room temperature with contact time typical to those found in short cycle wash programmes.	To ensure that disinfection can be achieved in DLMs that do not reach the requested programme temperature in the minimum contact time typically found in DLMs
If relying on thermal killing select cycle temperature >70°C	By targeting 70°C it is more likely that the temperature will at least reach >60°C for 10min	Offer a servicing and performance (temperature monitoring) check of healthcare workers DLM.	Ensuring that healthcare uniforms are being disinfected effectively.
Disinfect and clean DLM regularly with washing machine cleanser and/or by performing a very high temperature empty wash (>90 °C)	Cleaning the DLM regularly aids with reducing biofilm formation	Offer On Premise Laundering (OPL) with a regularly controlled washing machine (performance and biofilm formation) and efficient detergent	By offering OPL the employer will have control over the decontamination performance of healthcare uniforms in term of thermal and chemical disinfection
Do not mix the laundering of uniforms with any other garment.	Avoid the possibility of cross contamination	Outsource the laundering of healthcare uniforms to industrial launders accredited to BS EN 14065:2016. Textiles - Laundry processed textiles - Biocontamination control system [46]	Industrial laundries are obligated to check the performance of their laundering process and ensure disinfection is achieved
Renew the DLM regularly preferably every four years	DLM performance reduces over time and regular renewing of the DLM will ensure that the laundry is performed under optimal conditions	Source uniforms that can be laundered at temperatures at or above 60°C	A combination of thermal and chemical disinfection provides more effective cleaning for healthcare uniforms, but it can only be applied if the uniform’s textile can withstand the process without damage
		If laundry is performed on-site without performance controls in place, the DLM should be renewed regularly, ideally every four years.	DLM performance reduces over time and regular renewing of the DLM will ensure that the laundry is performed in optimal conditions

By implementing these measures, healthcare settings can reduce the risk of infection transmission and the potential increased antibiotic resistance associated with inadequate laundering of healthcare uniforms. Ensuring the thorough disinfection of healthcare uniforms is crucial for protecting both patients and healthcare workers from avoidable infections.

## Supporting information

S1 Fig Schematic representation of the bioinformatic analysis performed on the DLM shotgun metagenomic sequences. (TIF)

S2 Fig Temperature profile of DLMs during short and standard wash cycles. (TIF)

S1 Table *Pseudomonas aeruginosa* antibiotic susceptibility profile before and after long-term exposure to domestic detergent. (DOCX)

S2 Table *Klebsiella pneumoniae* antibiotic susceptibility profile before and after long-term exposure to domestic detergent. (DOCX)

S3 Table Staphylococcus aureus antibiotic susceptibility profile before and after long-term exposure to domestic detergent. (DOCX)

S1 File Supplementary methods. (DOCX)
