# Supplementary figures and images for: Domestic laundering of healthcare textiles: Disinfection efficacy and risks of antibiotic resistance transmission
Source: PLoS One. 2025 Apr 30;20(4):e0321467. doi: 10.1371/journal.pone.0321467 (PMC12043170; doi:10.1371/journal.pone.0321467)

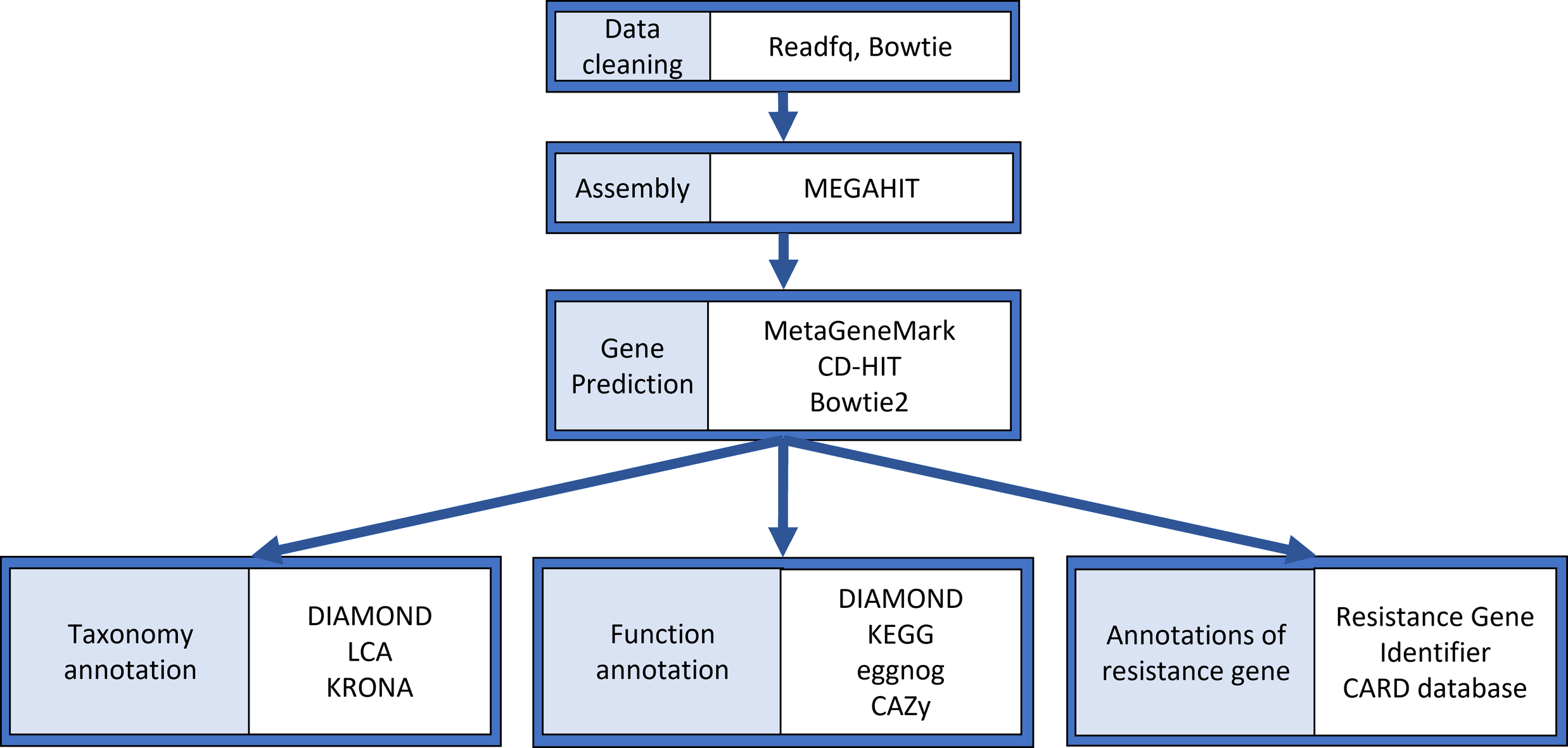

Supplement: S1 Fig — (TIF) [file pone.0321467.s001.tif]

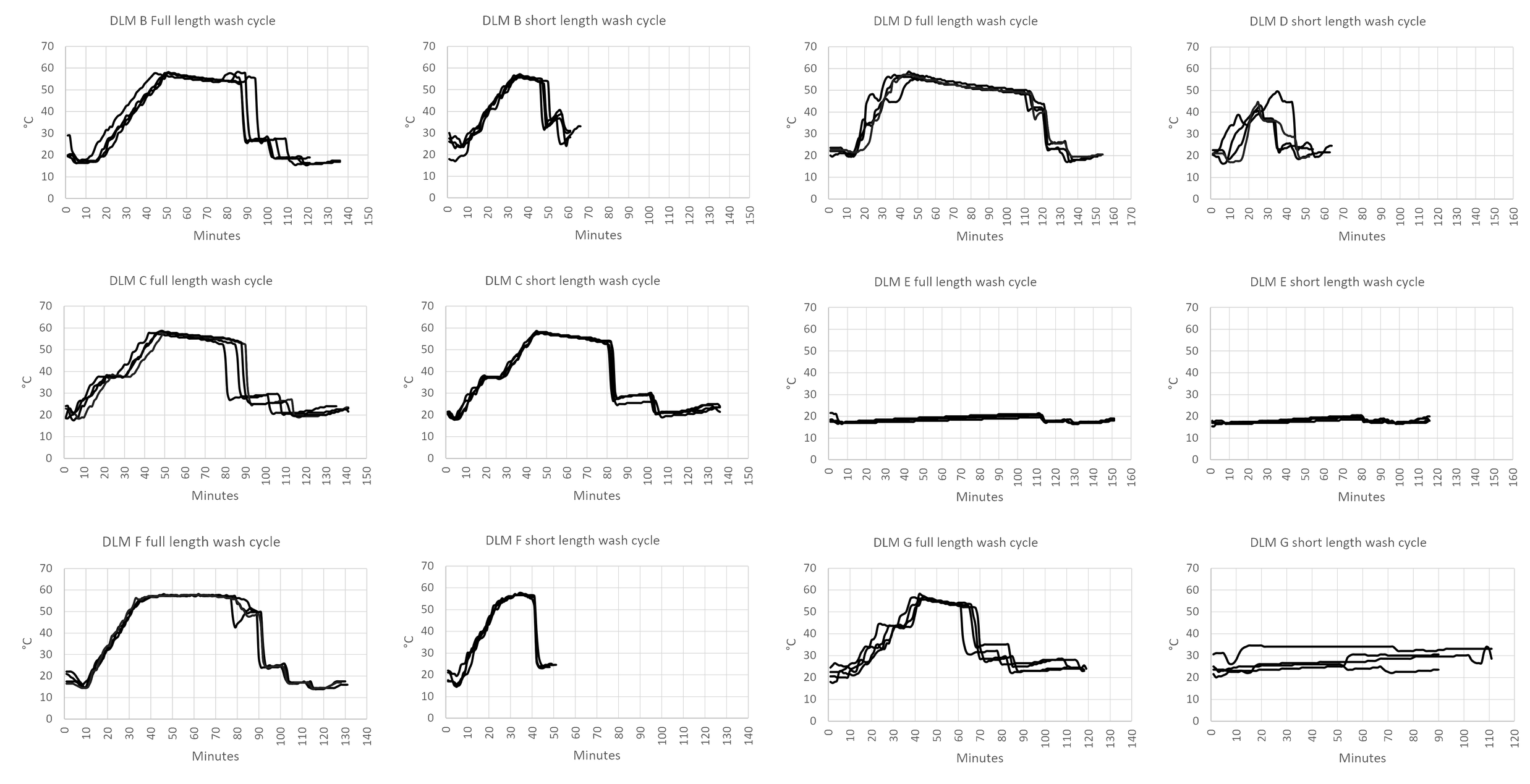

Supplement: S2 Fig — (TIF) [file pone.0321467.s002.tif]
