# Supplementary material for: Domestic laundering of healthcare textiles: Disinfection efficacy and risks of antibiotic resistance transmission
Source: PLoS One. 2025 Apr 30;20(4):e0321467. doi: 10.1371/journal.pone.0321467 (PMC12043170; doi:10.1371/journal.pone.0321467)
Supplement: S1 Table — (DOCX) [file pone.0321467.s003.docx]

| **Table S1. *Pseudomonas aeruginosa* antibiotic susceptibility profile before and after long-term exposure to domestic detergent.** | | | | | | | | | |
| --- | --- | --- | --- | --- | --- | --- | --- | --- | --- |
| **Test Phase** | **Antibiotic** | **Detergent Type** | **Zone of inhibition (mm)** | | | | | | **Difference pre and post exposure (mm)** |
|  |  |  | **Pre-detergent exposure** | | | **Post-detergent exposure** | | |  |
|  |  |  | **Mean** | **SD** | **Resistance Status*** | **Mean** | **SD** | **Resistance Status*** |  |
| **Clinically relevant antibiotic screen** | Ciprofloxacin 5μg | Liquid | 28.9 | 0.22 | S | 27.63 | 0.23 | S | 1.27 |
|  | Ceftazidime 10μg |  | 18.59 | 0.05 | S | 19.59 | 0.17 | S | -1 |
|  | Meropenem 10μg |  | 30.76 | 0.29 | S | 28.46 | 0.13 | S | 2.3 |
|  | Amikacin 30μg |  | 19.41 | 0.33 | S | 18.37 | 0.1 | S | 1.04 |
|  | Aztreonam 30μg |  | 20.9 | 0.03 | S | 20.23 | 0.31 | S | 0.67 |
|  | Ciprofloxacin 5μg | Powder | 28.9 | 0.22 | S | 27.93 | 0.48 | S | 0.97 |
|  | Ceftazidime 10μg |  | 18.59 | 0.05 | S | 18.39 | 0.51 | S | 0.2 |
|  | Meropenem 10μg |  | 30.76 | 0.29 | S | 29.79 | 0.49 | S | 0.97 |
|  | Amikacin 30μg |  | 19.41 | 0.03 | S | 18.2 | 0.09 | S | 1.21 |
|  | Aztreonam 30μg |  | 20.9 | 0.03 | S | 19.79 | 0.15 | S | 1.11 |
| **M14 Ring**  **Screen** | Colistin Sulphate 25µg | Liquid | 16.09 | 0.32 | N/A | 14.39 | 0.18 | N/A | 1.64 |
|  | Gentamicin 10µg |  | 19.72 | 0.36 | N/A | 17.14 | 0.42 | N/A | 2.58 |
|  | Streptomycin 10µg |  | 11.48 | 0.4 | N/A | 7.77 | 0.16 | N/A | 3.71 |
|  | Tetracycline 25µg |  | 7.74 | 0.32 | N/A | 4.65 | 3.29 | N/A | 3.09 |
|  | Colistin Sulphate 25µg | Powder | 16.09 | 0.32 | N/A | 14.8 | 0.17 | N/A | 2.1 |
|  | Gentamicin 10µg |  | 19.72 | 0.36 | N/A | 17.28 | 0.43 | N/A | 2.44 |
|  | Streptomycin 10µg |  | 11.48 | 0.4 | N/A | 7.48 | 0.52 | N/A | 4 |
|  | Tetracycline 25µg |  | 7.74 | 0.32 | N/A | 6.57 | 0.33 | N/A | 1.17 |

N/A= Lack of a EUCAST breakpoint for this antibiotic. That may be due to dosage, or due to the antibiotic not being a recommended clinical option.

*Based on EUCAST breakpoints (EUCAST, 2024)
