# Supplementary material for: Domestic laundering of healthcare textiles: Disinfection efficacy and risks of antibiotic resistance transmission
Source: PLoS One. 2025 Apr 30;20(4):e0321467. doi: 10.1371/journal.pone.0321467 (PMC12043170; doi:10.1371/journal.pone.0321467)
Supplement: S2 Table — (DOCX) [file pone.0321467.s004.docx]

| **Table S2. *Klebsiella pneumoniae* antibiotic susceptibility profile before and after long-term exposure to domestic detergent.** | | | | | | | | | |
| --- | --- | --- | --- | --- | --- | --- | --- | --- | --- |
| **Test Phase** | **Antibiotic** | **Detergent Type** | **Zone of inhibition (mm)** | | | | | | **Difference pre and post exposure (mm)** |
|  |  |  | **Pre-detergent exposure** | | | **Post-detergent exposure** | | |  |
|  |  |  | **Mean** | **SD** | **Resistance status** | **Mean** | **SD** | **Resistance status** |  |
| **Clinically relevant antibiotic screen** | Ertapenem 10μg | Liquid | 29.56 | 0.24 | S | 28.23 | 0.4 | S | 1.33 |
|  | Meropenem 10μg |  | 28.1 | 0.12 | S | 27.58 | 0.29 | S | 0.52 |
|  | Ciprofloxacin 5μg |  | 33.15 | 0.53 | S | 32.19 | 0.5 | S | 0.96 |
|  | Moxifloxacin 5μg |  | 30.14 | 0.45 | S | 30.86 | 0.25 | S | -0.72 |
|  | Amikacin 30μg |  | 19.08 | 0.25 | S | 18.76 | 0.43 | S | 0.32 |
|  | Cefpoxidime 10μg |  | 29.56 | 0.43 | S | 28.06 | 0.34 | S | 1.5 |
|  | Ceftazidime 10μg |  | 25.05 | 0.19 | S | 23.51 | 0.62 | S | 1.54 |
|  | Aztreonam 30μg |  | 29.23 | 0.15 | S | 29.38 | 0.11 | S | -0.15 |
|  | Ertapenem 10μg | Powder | 29.56 | 0.24 | S | 19.18 | 0.39 | R | 10.38 |
|  | Meropenem 10μg |  | 28.1 | 0.12 | S | 13.98 | 0.38 | R | 14.12 |
|  | Ciprofloxacin 5μg |  | 33.15 | 0.53 | S | 28.75 | 0.61 | S | 4.4 |
|  | Moxifloxacin 5μg |  | 30.14 | 0.45 | S | 25.81 | 0.32 | S | 4.33 |
|  | Amikacin 30μg |  | 19.08 | 0.25 | S | 19.25 | 0.05 | S | -0.17 |
|  | Cefpoxidime 10μg |  | 29.56 | 0.43 | S | 25.11 | 0.90 | S | 4.45 |
|  | Ceftazidime 10μg |  | 25.05 | 0.19 | S | 23.83 | 0.35 | S | 1.22 |
|  | Aztreonam 30μg |  | 29.23 | 0.15 | S | 30.92 | 0.83 | S | -1.69 |
| **M14 Ring**  **Screen** | Ampicillin 10µg | Liquid | 7.41 | 0.18 | R | 0 | 0 | R | 7.41 |
|  | Cephalothin 5µg |  | 21.85 | 0.86 | N/A | 19.09 | 0.33 | N/A | 2.76 |
|  | Colistin Sulphate 25µg |  | 15.25 | 0.2 | N/A | 14 | 0.12 | N/A | 1.25 |
|  | Gentamicin 10µg |  | 21.56 | 0.44 | S | 19.03 | 0.01 | S | 2.53 |
|  | Streptomycin 10µg |  | 18.51 | 0.4 | N/A | 16 | 0.2 | N/A | 2.51 |
|  | Sulphatriad 200µg |  | 18.73 | 0.39 | N/A | 15.96 | 0.08 | N/A | 2.77 |
|  | Tetracycline 25µg |  | 27.25 | 0.22 | N/A | 24.99 | 0.52 | N/A | 2.26 |
|  | Cotrimoxazole 25µg |  | 27.45 | 0.51 | N/A | 24.76 | 0.13 | N/A | 2.69 |
|  | Ampicillin 10µg | Powder | 7.41 | 0.18 | R | 7.04 | 0.20 | R | 0.37 |
|  | Cephalothin 5µg |  | 21.85 | 0.86 | N/A | 16.30 | 0.79 | N/A | 5.55 |
|  | Colistin Sulphate 25µg |  | 15.25 | 0.2 | N/A | 14.56 | 0.24 | N/A | 0.69 |
|  | Gentamicin 10µg |  | 21.56 | 0.44 | S | 20.74 | 0.56 | S | 0.82 |
|  | Streptomycin 10µg |  | 18.51 | 0.4 | N/A | 16.09 | 0.20 | N/A | 2.42 |
|  | Sulphatriad 200µg |  | 18.73 | 0.39 | N/A | 20.26 | 0.74 | N/A | -1.63 |
|  | Tetracycline 25µg |  | 27.25 | 0.22 | N/A | 22.23 | 0.43 | N/A | 5.02 |
|  | Cotrimoxazole 25µg |  | 27.45 | 0.51 | N/A | 24.39 | 0.25 | N/A | 3.06 |

N/A= Lack of a EUCAST breakpoint for this antibiotic. That may be due to dosage, or due to the antibiotic not being a recommended clinical option.

*Based on EUCAST breakpoints (EUCAST, 2024)
