# Supplementary material for: Domestic laundering of healthcare textiles: Disinfection efficacy and risks of antibiotic resistance transmission
Source: PLoS One. 2025 Apr 30;20(4):e0321467. doi: 10.1371/journal.pone.0321467 (PMC12043170; doi:10.1371/journal.pone.0321467)
Supplement: S3 Table — (DOCX) [file pone.0321467.s005.docx]

| **Table S3. *Staphylococcus aureus* antibiotic susceptibility profile before and after long-term exposure to domestic detergent** | | | | | | | | | |
| --- | --- | --- | --- | --- | --- | --- | --- | --- | --- |
| **Test Phase** | **Antibiotic** | **Detergent Type** | **Zone of inhibition (mm)** | | | | | | **Difference pre and post exposure (mm)** |
|  |  |  | **Pre-detergent exposure** | | | **Post**-**detergent exposure** | | |  |
|  |  |  | **Mean** | **SD** | **Resistance Status*** | **Mean** | **SD** | **Resistance Status*** |  |
| **Clinically relevant antibiotic screen** | Cefoxitin 30μg | Liquid | 26.00 | 0.33 | S | 24.68 | 0.10 | S | 1.32 |
|  | Ciprofloxacin 5μg |  | 24.98 | 0.61 | S | 23.83 | 0.63 | S | 1.15 |
|  | Moxifloxacin 5μg |  | 25.77 | 0.19 | S | 24.87 | 0.44 | R | 0.9 |
|  | Erythromycin 15μg |  | 23.38 | 0.28 | S | 22.55 | 0.03 | S | 0.83 |
|  | Tetracycline 30μg |  | 22.37 | 0.12 | S | 23.19 | 0.45 | S | -0.82 |
|  | Linezolid 10μg |  | 20.34 | 0.47 | S | 21.27 | 0.34 | S | -0.93 |
|  | Fusidic Acid 10μg |  | 25.48 | 0.28 | S | 23.65 | 0.64 | R | 1.83 |
|  | Rifampicin 5μg |  | 26.40 | 0.05 | S | 26.06 | 0.27 | S | 0.34 |
|  | Vancomycin 30μg |  | 15.34 | 0.09 | S | 15.02 | 0.23 | S | 0.32 |
|  | Cefoxitin 30μg | Powder | 25.99 | 0.33 | S | 28.36 | 1.01 | S | -2.37 |
|  | Ciprofloxacin 5μg |  | 24.98 | 0.61 | S | 33.89 | 0.90 | S | -8.91 |
|  | Moxifloxacin 5μg |  | 25.77 | 0.19 | S | 34.38 | 0.84 | S | -8.61 |
|  | Erythromycin 15μg |  | 23.38 | 0.28 | S | 29.94 | 0.18 | S | -6.56 |
|  | Tetracycline 30μg |  | 22.37 | 0.12 | S | 18.16 | 0.31 | R | 4.21 |
|  | Linezolid 10μg |  | 20.34 | 0.47 | R | 29.77 | 0.71 | S | -9.43 |
|  | Fusidic Acid 10μg |  | 25.48 | 0.28 | S | 27.27 | 0.85 | S | -1.79 |
|  | Rifampicin 5μg |  | 26.40 | 0.05 | S | 18.67 | 0.50 | R | 7.73 |
|  | Vancomycin 30μg |  | 15.34 | 0.09 | S | 18.56 | 0.78 | S | -3.22 |
| **M13 Ring**  **Screen** | Chloramphenicol 25µg | Liquid | 28.62 | 0.29 | N/A | 18.9 | 0.98 | N/A | 9.72 |
|  | Erythromycin 5µg |  | 28.78 | 1.21 | N/A | 18.59 | 0.75 | N/A | 10.19 |
|  | Fusidic acid 10µg |  | 33.28 | 0.55 | S | 26.28 | 1.02 | S | 7 |
|  | Oxacillin 5µg |  | 26.27 | 0.34 | S | 22.47 | 0.61 | S | 3.8 |
|  | Novobiocin 5µg |  | 30.71 | 0.83 | N/A | 27.01 | 0.75 | N/A | 3.7 |
|  | Penicillin G 1 unit |  | 29.08 | 0.58 | S | 24.76 | 1.31 | R | 4.32 |
|  | Streptomycin 10µg |  | 19.33 | 0.8 | N/A | 16.52 | 0.89 | N/A | 2.81 |
|  | Tetracycline 25µg |  | 33.71 | 0.70 | N/A | 27.05 | 0.55 | N/A | 6.66 |
|  | Chloramphenicol 25µg | Powder | 28.62 | 0.29 | N/A | 22.44 | 0.77 | N/A | 6.18 |
|  | Erythromycin 5µg |  | 28.78 | 1.21 | N/A | 25.22 | 1.69 | N/A | 3.56 |
|  | Fusidic acid 10µg |  | 33.28 | 0.55 | S | 32.31 | 0.62 | S | 0.97 |
|  | Oxacillin 5µg |  | 26.27 | 0.34 | S | 19.55 | 0.45 | R | 6.72 |
|  | Novobiocin 5µg |  | 30.71 | 0.83 | N/A | 32.99 | 0.81 | N/A | -2.28 |
|  | Penicillin G 1 unit |  | 29.08 | 0.58 | S | 23.33 | 1.53 | R | 5.75 |
|  | Streptomycin 10µg |  | 19.33 | 0.80 | N/A | 17.77 | 0.62 | N/A | 1.56 |
|  | Tetracycline 25µg |  | 33.71 | 0.70 | N/A | 34.1 | 0.91 | N/A | -0.39 |

**N/A= Lack of a EUCAST breakpoint for this antibiotic. That may be due to dosage, or due to the antibiotic not being a recommended clinical option.**

*Based on EUCAST breakpoints (EUCAST, 2024)
