# Supplementary material for: Domestic laundering of healthcare textiles: Disinfection efficacy and risks of antibiotic resistance transmission
Source: PLoS One. 2025 Apr 30;20(4):e0321467. doi: 10.1371/journal.pone.0321467 (PMC12043170; doi:10.1371/journal.pone.0321467)
Supplement: S1 File — (DOCX) [file pone.0321467.s006.docx]

**Supplementary methods**

1. **Shotgun metagenomic sequencing and data analysis**

Among the 24 samples, 12 samples (From eight DLMs) which exhibited sufficient DNA concentrations (> 10ng/µl) were analysed by shotgun metagenomic sequencing. The Shotgun metagenomic sequencing was performed by Novogene Company Ltd. (UK) using the PE150 strategy and the Illumina NovaSeq 6000 S4 platform following the manufacturer instruction and Novogene internal optimisation. The sequencing data analysis included taxonomic, function and antibiotic resistance gene annotations and was performed by Novogene following the pipeline summarised in Fig S1.

The ten most common microbial classes, genera and species were identified to determine the general microbiome profile of the DLM.

The relative abundance of genera containing bacteria known to be resistant to antibiotics or with increasing antibiotic resistance and responsible for nosocomial infections was specifically extracted from the data. The bacteria genera specifically targeted were *Acinetobacter, Bacillus, Citrobacter, Clostridioides, Enterobacter, Enterococcus, Escherichia, Klebsiella, Mycobacterium, Pseudomonas, Staphylococcus* and *Streptococcus*.

1. **Sublethal concentrations of domestic laundry detergent**

Two commonly used UK washing detergents were selected for the assay: a non-biological liquid detergent (15-30%:Anionic surfactants; 5-15%:nonionic surfactants; <5%:phosphonate, perfume, soap, optical brighteners, methylisothiazolinone, octylisothiazolinone) and a non-biological powder detergent (5-15%: oxygen-based bleaching agents, anionic surfactants; <5%: nonionic surfactants, polycarboxylates, soap, perfume, phosphonates, optical brighteners, zeolites). Sublethal concentrations of domestic laundry detergents were determined by calculating 80% inhibition of growth, this was determined using the microtitration method. Domestic non-biological washing detergents solutions were mixed at a 1:1 ratio with a bacteria solution adjusted to OD_600nm_ of 0.2 (10^8^ CFU/ml) to reach the final detergent concentrations ranging from 0.675µl/ml to 0.00000675µl/ml for the liquid detergent and from 0.18g/ml to 0.000018g/ml for the powder detergent. A positive control consisting of a 1:1 ratio of nutrient broth and the bacteria adjusted to OD_600nm_ of 0.2 was included, as well as negative controls consisting of nutrient broth only or a 1:1 ratio of nutrient broth with each detergent. The optical density at 600nm was measured using a colorimeter (WPA C0700B) before and after incubation at 37°C for 24h in aerobic conditions.

1. **Whole Genome Sequencing of antibiotic cross-resistant strains**

The original *S. aureus* NCTC 10788 strain, two DLM detergent tolerant mutant *S. aureus* strains isolated after 15 passages, the original *K. pneumoniae* NCIMB 10341 strain, and one DLM detergent tolerant mutant *K. pneumoniae* strain isolated after 15 passages in the powder detergent were analysed by whole genome sequencing (WGS). The bacteria strains were cultured on nutrient agar at 37°C for 24h in aerobic conditions. After incubation the bacteria cells were collected and genomic DNA was extracted using the FastDNA soil spin kit (MP biomedicals, Germany). WGS was performed by Novogene Company Ltd (UK) using the Illumina NovaSeq 6000 S4 platform for the PE150 sequencing strategy.

The genomes of the S*. aureus* strains and *K. pneumoniae* strains isolated before and after the laundry detergent tolerance induction assay were compared to identify any genome modifications using Snippy (version 4.6.0) [1].

**Reference.**

1. Seemann T. Snippy: fast bacterial variant calling from NGS reads. 2015. Available from: https://github.com/tseemann/snippy.
